# GAIA Museum - a special place in Denmark featuring artist Maria Sloth Sørensen

**DOI:** 10.1017/S2045796023000215

**Published:** 2023-05-03

**Authors:** Anna Noe Bovin

**Affiliations:** GAIA Museum, Randers, Denmark

## GAIA Museum – a special place in Denmark

GAIA Museum Outsider Art was founded in Randers in 2002. It is a special museum with a focus on outsider art, also called Art Brut. GAIA is showing the breadth of outsider art in a permanent exhibition as well as in changing special exhibitions. It differs from other museums by having creative workshops, a studio and art academy. The organization is run by artistic and pedagogical educated staff together with people with various handicaps, such as Downs Syndrome, Autism and intellectual disabilities.

The collection at GAIA Museum consists of works from all over the world. New works of art are added, often in the form of donations, or in connection with the museum’s three annual special exhibitions, where selected works are purchased and included in the collection (Bovin, 2021; GAIA Staff, 2022a).

At GAIA Museum, outsider art is defined as art outside the mainstream, often created by people with physical, mental or social disabilities. But most of all with the British art professor Roger Cardinal’s description:

“Outsider art is the product of an authentic impulse to create and is free of conscious artifice. It has nothing to do with contrivance and academic standards, everything to do with passion and caprice” (Roger Cardinal, 1979)

## The GAIA academy of arts

In connection with the museum is GAIA Academy, which is a studio and art education whose purpose is to support the artists’ work and development. The working day alternates between joint projects and own work. The artists are periodically introduced to different working methods and techniques, and inspiration is drawn from museum visits, travels or visits from guests who come to GAIA. The Academy Artists not only work with art on a practical, physical level but also with the dissemination of art, for example, with the awareness of their own works and the development of the ability to present their own art to others.

This article presents an artist who in her work reflects a lot on both the current events in the world and on her own life.

**Figure 1.**
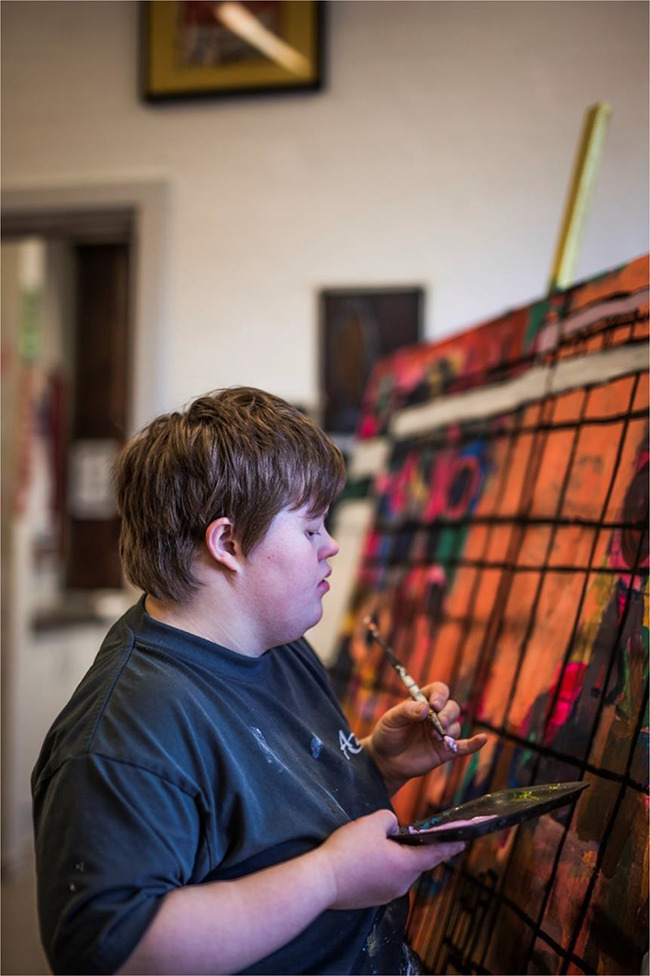
Maria Slot Sørensen painting at GAIA (Photo Esteban Buhrkall).

Maria Sloth Sørensen was born in Denmark in 1989. She is a painter and has been at GAIA Academy since 2012, where she has developed a personal style and way of expression that is characterized by strong lines and bright colours (Figure 1).

**Figure 2.**
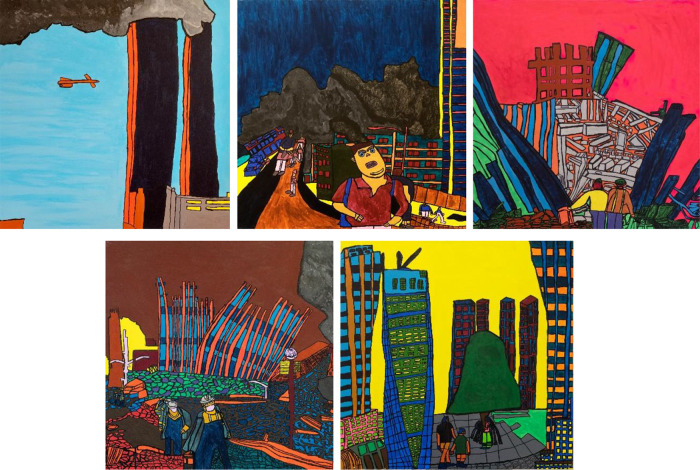
The five painting in Maria’s 9.11 series.

Maria tackles serious themes and current events in her works. For example, she has created an impressive series of five large paintings about the terrorist attack in New York on September 11, 2001 (Figure 2), about the tsunami in the Indian Ocean on December 26, 2004, and about the terrorist action at the Powder Barrel in Copenhagen on December 11, 2015. Recently, she has reflected on the COVID crises and lock downs in her artwork (Bovin, 2022; GAIA Staff, 2022b).

A distinctive feature of Maria’s art is that she often incorporates text directly into the motif or alternatively writes on the back of the canvases. In these texts, Maria’s reflections on life and the world become accessible. It adds an extra layer to the artwork and gives the audience a chance to get more insight into Maria’s thoughts and reflections.

**Figure 3.**
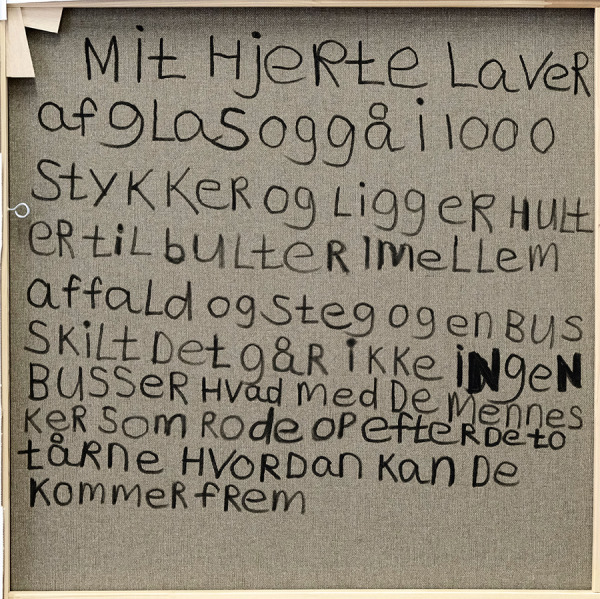
‘My heart is made of glass’. Maria often writes on the backsides of her paintings. Here the text from the fourth painting in the 9.11 series.

Here is the back of the fourth painting in the 9.11 series. Maria describes how it feels as if her heart is made of glass and breaks into a thousand pieces and lies hollow among the debris. She notices that there is a bus sign, but the buses do not run. So, what about the people who have to clean up after the demolition of the two towers. How will they get there? (Figure 3)

**Figure 4.**
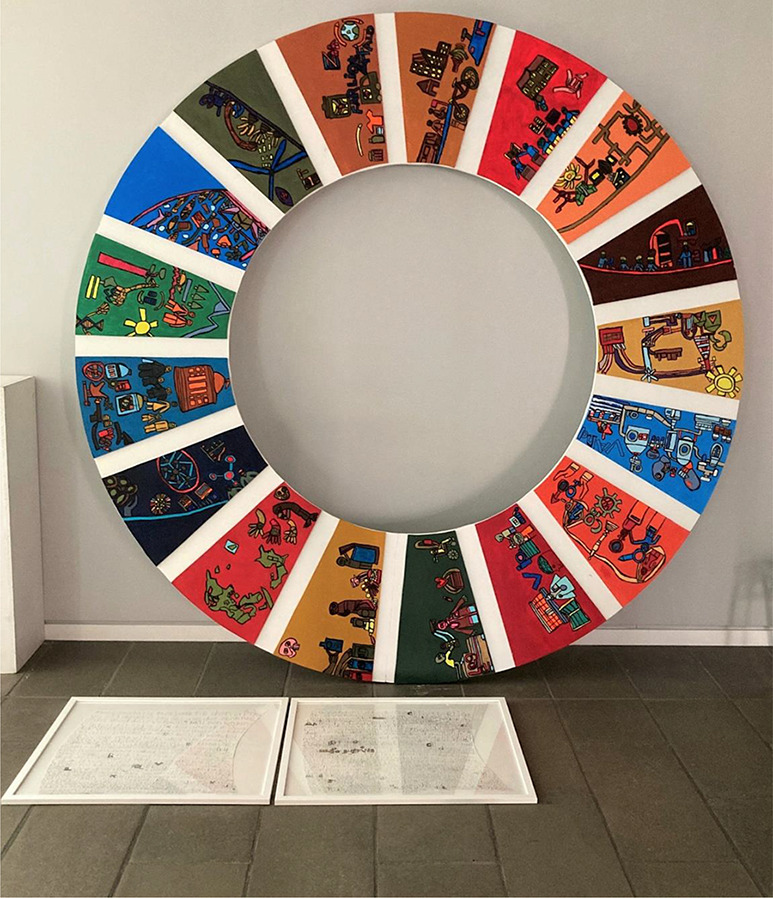
UN’s 17 World Goals interpreted by Maria.

In later works, Maria has been inspired by and worked with the UN’s 17 World Goals. Among other things, she has created this installation, where she reproduces the 17 world goals in a circle that measures 2.5 × 2.5 meters. The installation is on display in Randers Municipality’s administration building and has been part of a large world goal festival in April 2022 (Figure 4).

Maria’s reflections are included in two framed texts that belong to the work. She writes, among other things, that it is important to take care of nature and the environment and that there are different approaches in different countries, as conditions are not the same in the world. Maria writes a lot about COVID because the lock downs of society affected her a lot while she was working on the project. She was particularly affected by not being able to go to work at GAIA Academy, to music festivals and other social events or travel – and she mentions in the text how much she is looking forward to getting her normal everyday life back. Maria has seen it as her mission to carry out the creation of the work, so that it was ready for exhibition when the worst corona crisis was over.

Many of Maria’s works are privately owned, as the GAIA Academy also serves as a gallery for its artists. But some can be seen in various public buildings, including Randers Municipality’s administration building on Laksetorvet, Region Hospital Randers and VIA University College – Campus Randers and of course at GAIA.

Maria masters both the very large formats in painting, but she also masters portrait drawings. Among other things, she has made a whole series of ink drawings of the people she works with. The line is intense and alive, and like Maria’s other works, they possess an expressiveness that rarely fails to affect the viewer.

## Interview with Maria

Can you tell us a little about yourself, your upbringing and your life?

I am 35 years old. I live at a sheltered housing, Marienborgvej, in Randers. I grew up with my mother and father and two younger brothers. I am married to my husband, Casper. I go to many activities in my spare time, i.e., football and swimming.

What do you think about being an artist?

I am happy to be an artist. When other people see my art and appreciate it, I feel proud. I am happy that I can make art every day, because I like it. I like to draw and paint.

What is important to you?

Every time I have made a painting, there is a text that goes with the painting. My paintings and texts help to explain how I feel, and that is important to me.

How do you choose your themes and motifs?

By listening to music. There I get different emotions in me. I also love drama. My art is also inspired by what is happening in society.

How long do you think about it before you start painting?

It takes one to two weeks from when I get an idea until I start drawing the motif.

Do you know in advance what you want to do and what it will look like?

**Figure 5.**
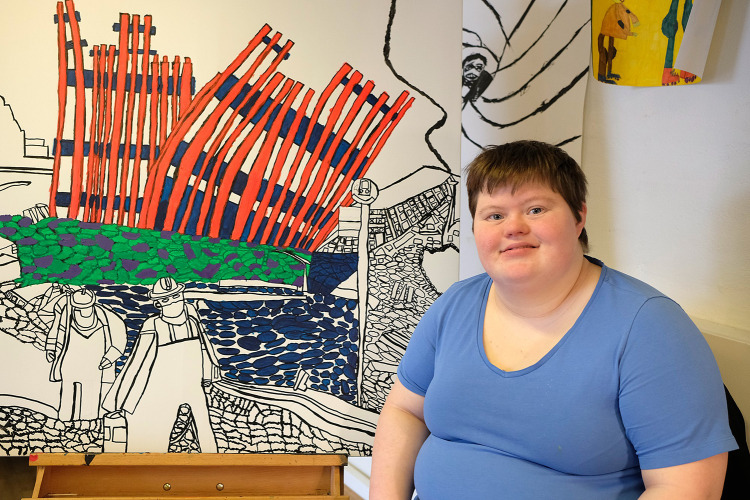
Maria in front of work in progress from the 9.11 series. Notice the technique of drawing up in pencil and marker and finally colouring.

I draw with a pencil first, and then with a marker. Then the ideas come to me about what colours to use (Figure 5).

When and why did you start writing on your pictures?

When you just see my picture, the text can help explain the picture and the thoughts behind it. I like that.

What are your future dreams?

I like to challenge myself and would like to paint paintings that are a bit difficult for me to paint. I would like to exhibit and sell my art. I would like to exhibit abroad.

Is there anything else you would like to tell the readers of the article?

I like it when guests at GAIA come by my workstation, and when they talk to me about what I’m doing.

## Supplementary material

For supplementary material accompanying this paper visit http://dx.doi.org/10.1192/bjb.2018.41. click here to view supplementary material
