# Supplementary figures and images for: GAIA Museum - a special place in Denmark featuring artist Maria Sloth Sørensen
Source: Epidemiol Psychiatr Sci. 2023 May 3;32:e30. doi: 10.1017/S2045796023000215 (PMC10227532; doi:10.1017/S2045796023000215)

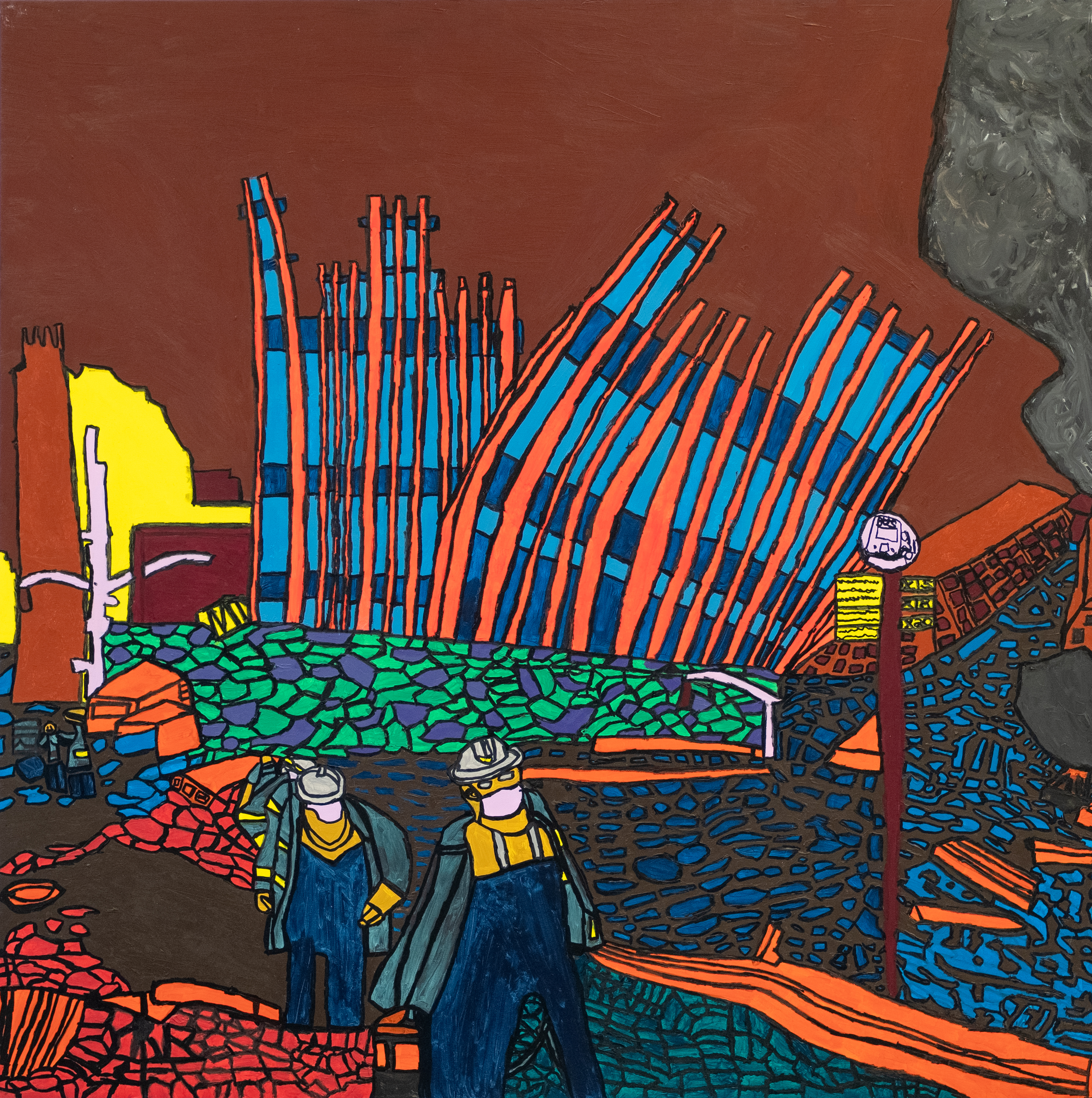

Supplement: Supplementary file 1 [file S2045796023000215sup001.jpg]
